# Supplementary material for: Unsupervised machine learning predicts future sexual behaviour and sexually transmitted infections among HIV-positive men who have sex with men
Source: PLoS Comput Biol. 2022 Oct 27;18(10):e1010559. doi: 10.1371/journal.pcbi.1010559 (PMC9642906; doi:10.1371/journal.pcbi.1010559)
Supplement: S1 Text — (PDF) [file pcbi.1010559.s001.pdf]

Unsupervised machine learning predicts future sexual behaviour and sexually transmitted infections among HIV-positive men who have sex with men

Andresen *et al.*, 2022

SUPPLEMENTARY MATERIAL

Section 1.

Software

All analyses were performed in R version 3.6.0 using the *stats* (version 3.6.0), *lmtest* (version 0.9-37), and *lme4* (version 1.1-21) packages. *stats*, *dendextend* (version 1.12.0) and *ape* (version 5.3) packages were used for clustering and *ggplot2* (version 3.1.1) and *ggthemes* (Economist colour palette; version 4.2.0) for data visualisation. All codes are available on github.com/Kouyos-Group/ Behavioural-Clusters-and-STIs.

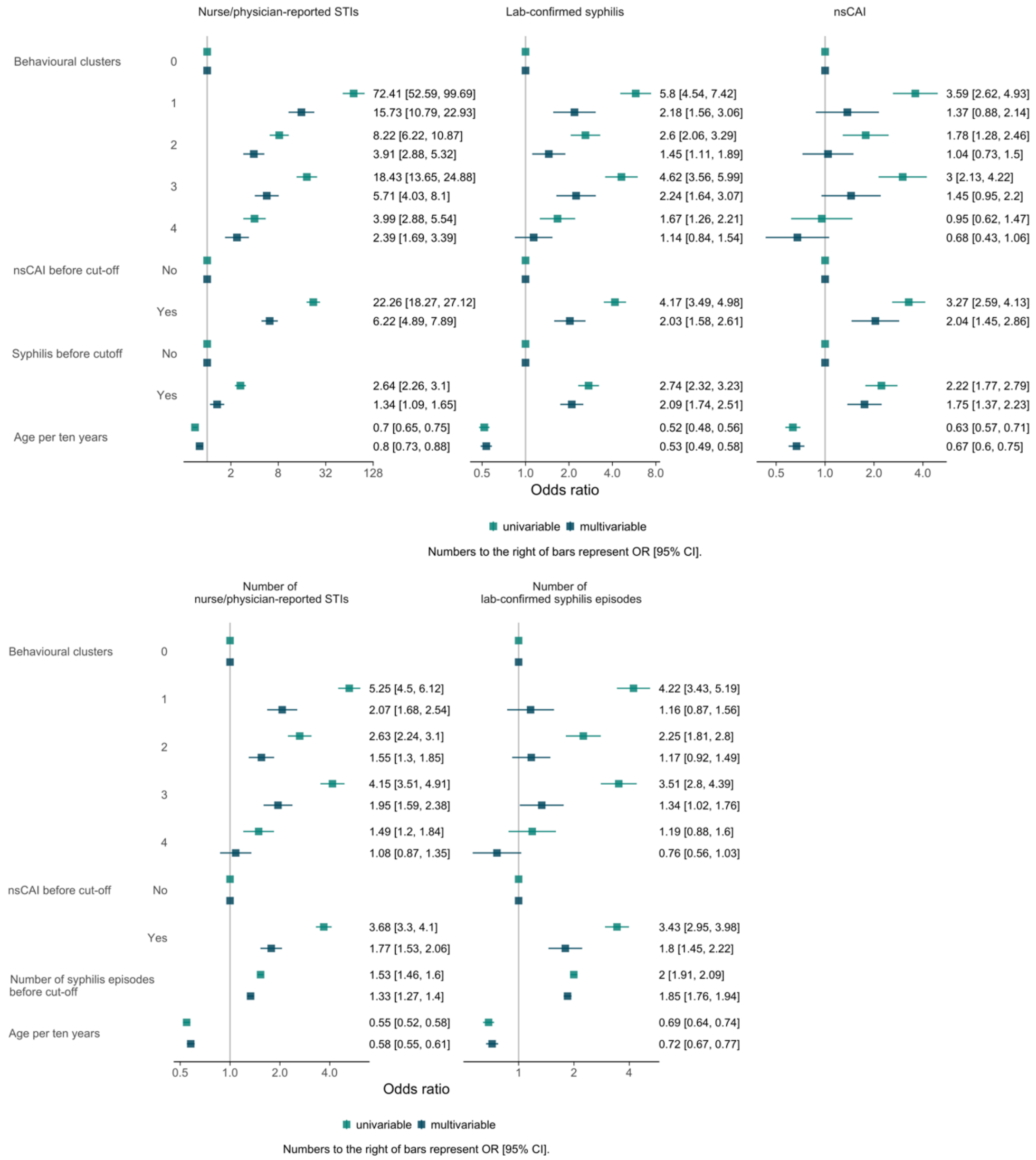

Fig A: Forest plots for factors associated with nurse/physician-reported STIs , laboratory-confirmed syphilis and nsCAI after cut-off. As well as number of nurse/physician-reported STIs and number of laboratory-confirmed syphilis episodes (bottom row). Odds ratios and 95% confidence intervals are shown. Behavioural clusters were inferred using data until the 1st of May 2017 (cut-off), and outcome variables were recorded after this date. nsCAI = condomless anal intercourse with non-steady partners. OR = odds ratio. CI = confidence interval.

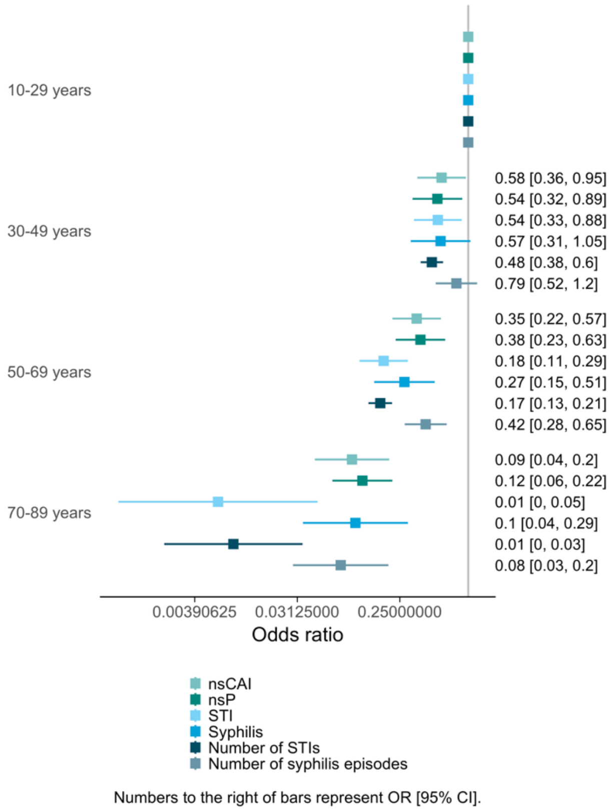

Fig B: Association between age and future sexual behaviour and STIs. Forest plot with odds ratio and 95% confidence interval for different outcomes for different age groups. Participants aged 10-29 years served as the reference group. nsCAI = condomless anal intercourse with non-steady partners. nsP = sex with non-steady partners. OR = odds ratio. CI = confidence interval.

a

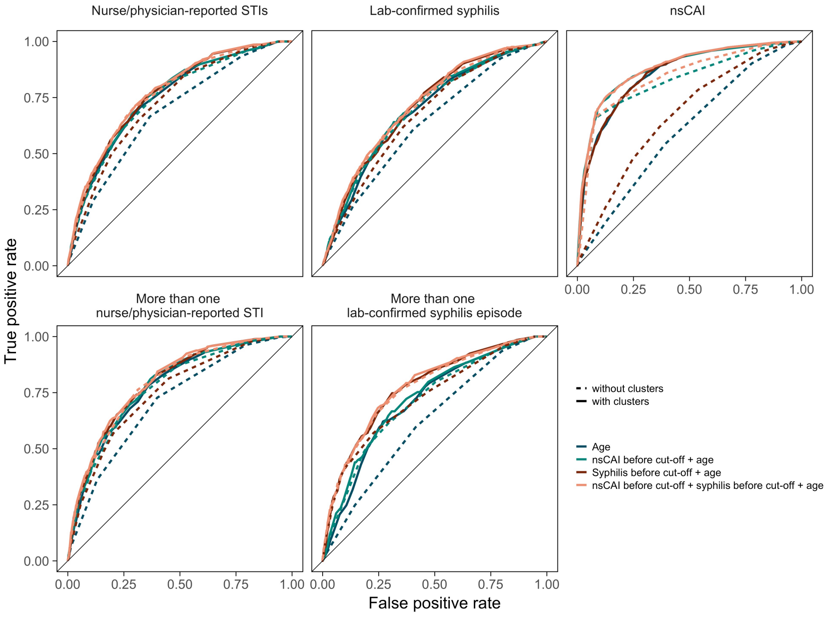

For repeated STI/syphilis outcomes (bottom row), syphilis before cut-off refers to more than one syphilis episode before cut-off.

b

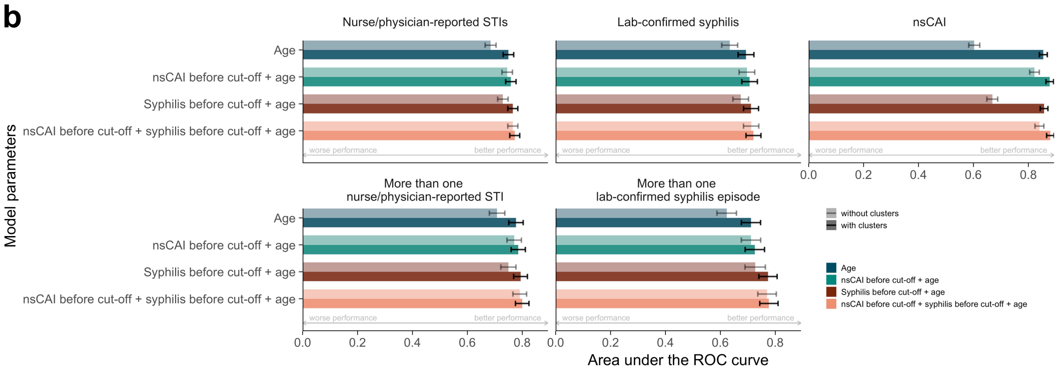

For repeated STI/syphilis outcomes (bottom row), syphilis before cut-off refers to more than one syphilis episode before cut-off.

Fig C: ROC curves and areas under the ROC curve for prediction models. **Panel a**: ROC curves for prediction of physician-reported STIs, laboratory-confirmed syphilis and nsCAI after cut-off as well as number of nurse/physician-reported STIs and laboratory-confirmed syphilis episodes after cut-off (bottom row).Colours show different model configurations, solid lines indicate models that additionally include clusters as a predictor. **Panel b**: areas under the curve of ROC shown in a. Error bars represent 95% confidence intervals. Colours show different model configurations, darker bars indicate models that additionally include clusters as a predictor. ROC = receiver operator characteristic. nsCAI = condomless anal intercourse with non-steady partners.

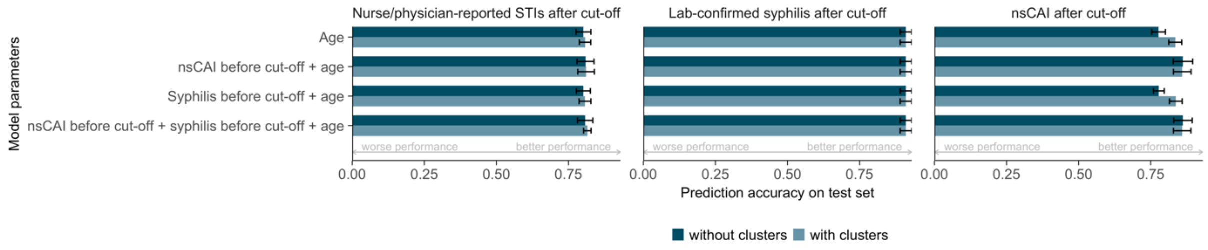

Fig D: Prediction accuracy on test set. Mean accuracy and 95% confidence interval after five-fold cross-validation are shown. nsCAI = condomless anal intercourse with non-steady partners.

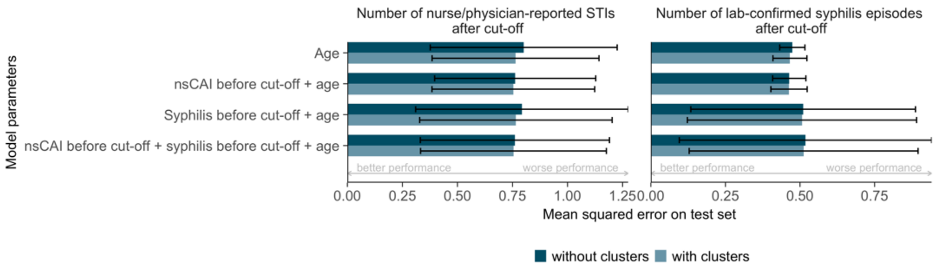

Fig E: Prediction error on test set. Mean of mean squared error and 95% confidence interval after five-fold cross-validation are shown. nsCAI = condomless anal intercourse with non-steady partners.

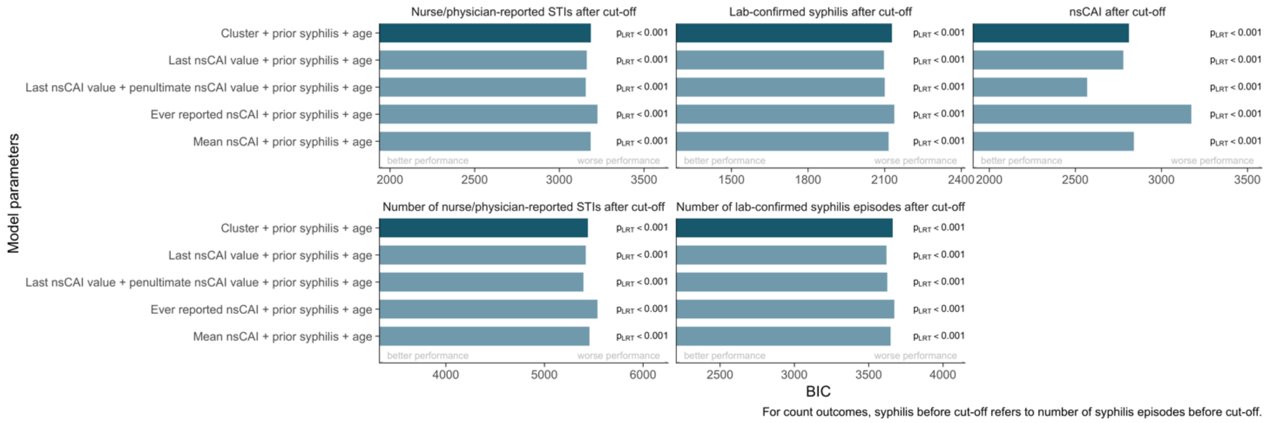

Fig F: BIC values for clusters versus other "past behaviour metrics". Model including behavioural clusters is highlighted in a darker shade. nsCAI = condomless anal intercourse with non-steady partners.

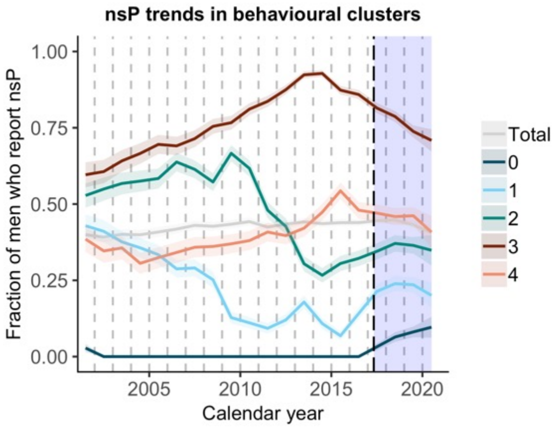

Fig G: Trends in nsP proportion in behavioural clusters. The clusters contain the following proportions of the study population (from 0 through 4): 14%, 18%, 19%, 30% and 19%. Cluster 0 consists of participants who never reported nsP during the observation period (in this case mid-2001 until the cut-off date). The dashed vertical line on the 1st of May 2017 represents the cut-off date between the observation period (which corresponds to the period used to infer the clusters) and outcome period. The shaded area shows the time in which nsP, STIs and syphilis were recorded as an outcomes. nsP = sex with non-steady partners.

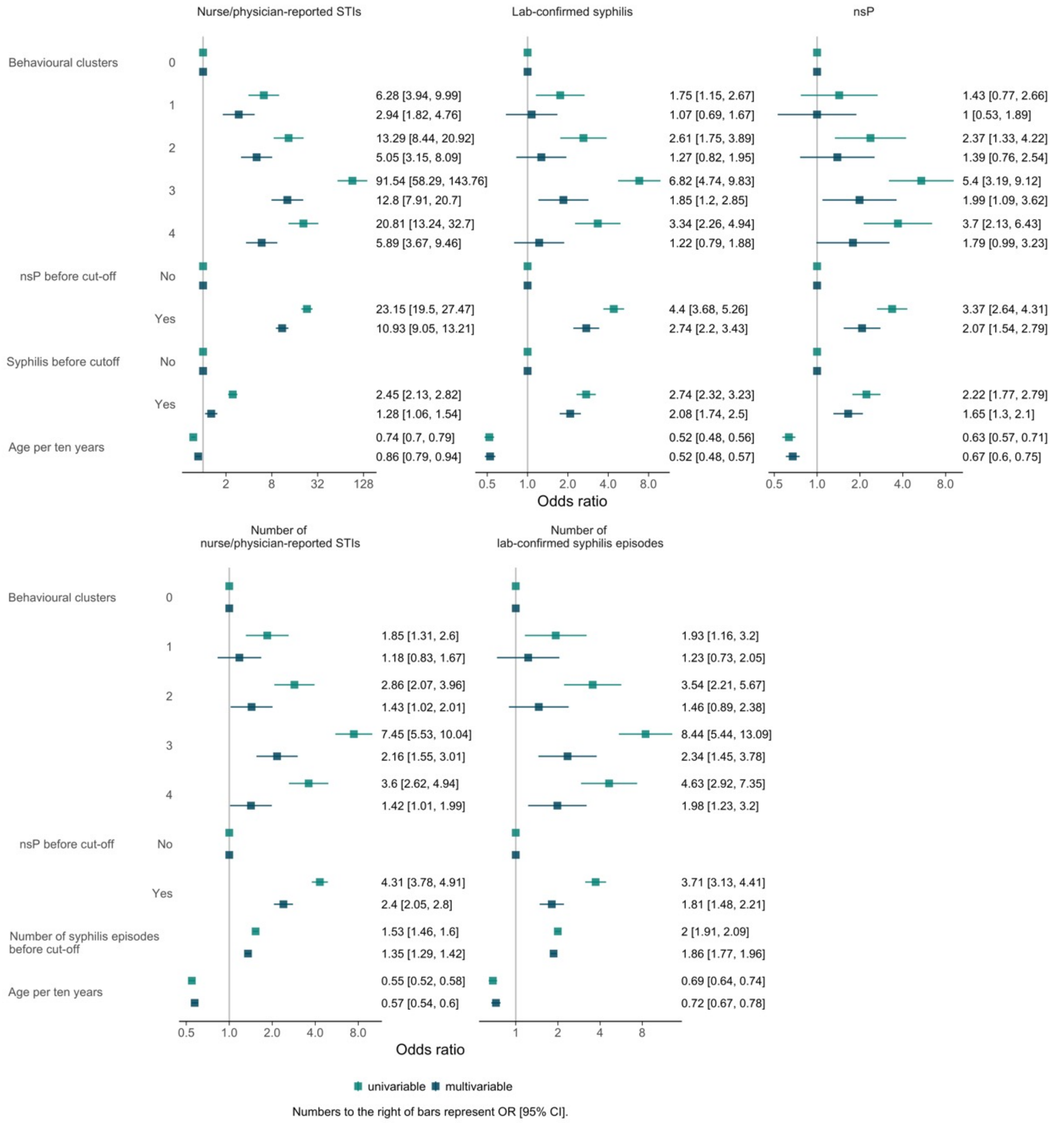

Fig H: Forest plots for factors associated with nurse/physician-reported STIs , laboratory-confirmed syphilis and nsP after cut-off. As well as number of nurse/physician-reported STIs and number of laboratory-confirmed syphilis episodes (bottom row). Odds ratios and 95% confidence intervals are shown. Behavioural clusters were inferred using data until the 1st of May 2017 (cut-off), and outcome variables were recorded after this date. nsP = sex with non-steady partners. OR = odds ratio. CI = confidence interval.

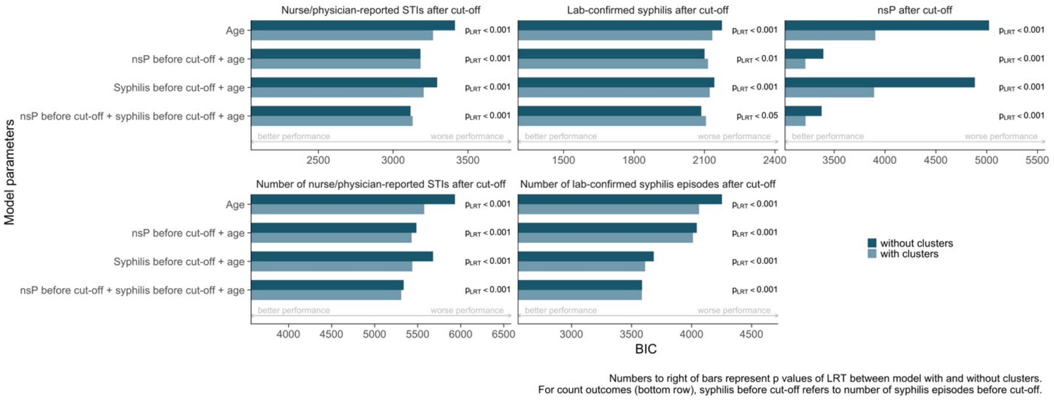

Fig I: Bar plots for Bayesian information criterion values. Regression models with different combinations of predictor variables to predict nurse/physician-reported STIs (left), laboratory-confirmed syphilis (center) and nsP (right) after cut-off. A smaller BIC represents a better prediction. Numbers to the right of the bars represent the p value of the likelihood ratio test between the two models in question. nsP = sex with non-steady partners. BIC = Bayesian information criterion. p_LRT = p value of likelihood ratio test comparing the respective models with and without clusters.

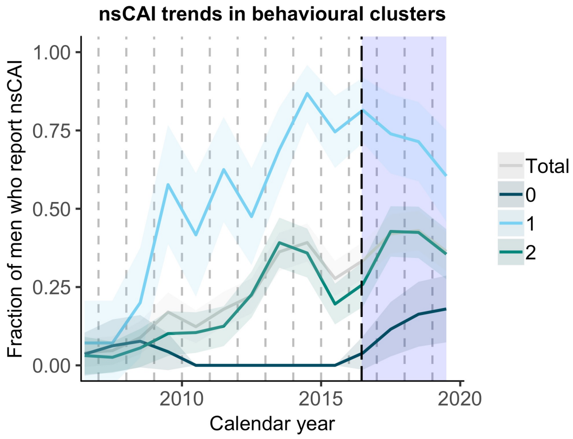

Fig J: Trends in nsCAI proportion in behavioural clusters. The clusters contain the following proportions of the study population (from 0 through 2): 19%, 24%, 57%. Cluster 0 consists of participants who never reported nsCAI during the observation period (in this case 2010 until mid-2016). The dashed vertical line on the 16/06/2016 represents the date until which data was used for clustering. The outcome (the number of sexual partners) was recorded in the period shaded in blue. nsCAI = condomless anal intercourse with non-steady partners.

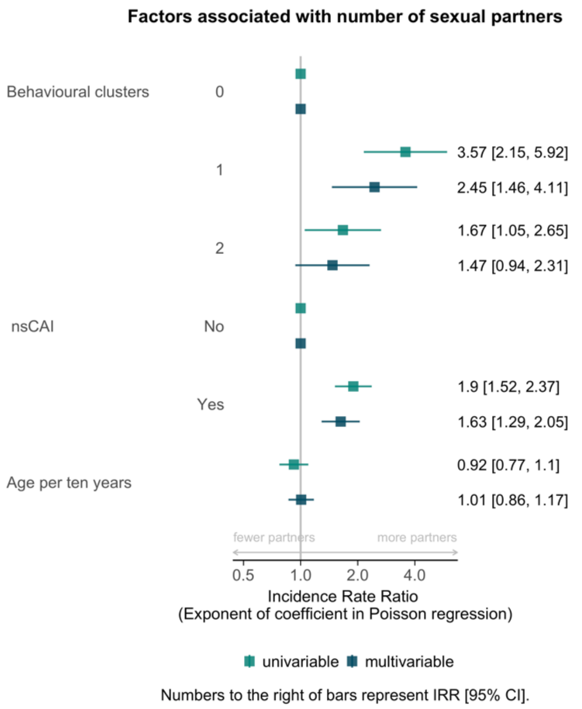

Fig K: Forest plot with factors associated with the number of self-reported sexual partners. Incidence rate ratios (IRR) and 95% confidence intervals (CI) are shown. IRR is the coefficient of Poisson regression and corresponds to the ratio between the incidence rates (thus here the "incidence" of having different sexual partners in 3 months) between groups. nsCAI = reported condomless anal intercourse with non-steady partners, reported at time of reporting number of sexual partners.

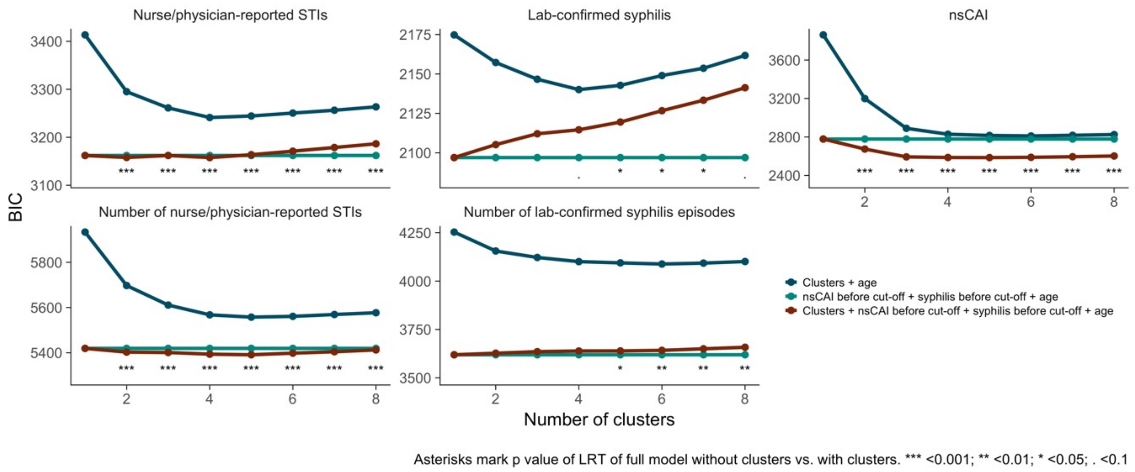

Fig L: BIC values for different numbers of clusters based on nsCAI trajectories. Prediction of nurse/physician-reported STIs, laboratory-confirmed syphilis (and number thereof, respectively, for count outcomes in bottom row), and nsCAI after cut-off. A lower BIC represents a better prediction. Asterisks mark the p-value of the likelihood ratio test between the second and third model. BIC = Bayesian information criterion, LRT = likelihood ratio test, nsCAI = condomless anal intercourse with non-steady partners.

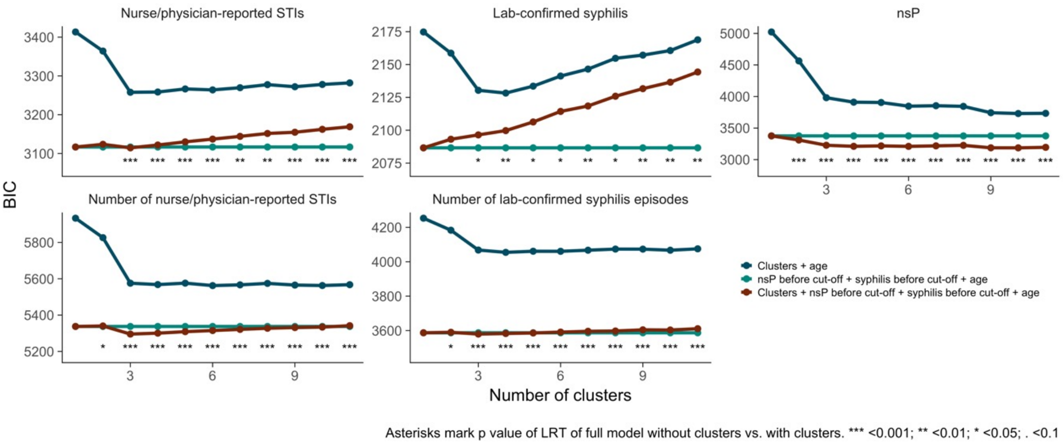

Fig M: BIC values for different numbers of clusters based on nsP trajectories. Prediction of nurse/physician-reported STIs, laboratory-confirmed syphilis (and number thereof, respectively, for count outcomes in bottom row), and nsP after cut-off. A lower BIC represents a better prediction. Asterisks mark the p-value of the likelihood ratio test between the second and third model. BIC = Bayesian information criterion, LRT = likelihood ratio test, nsP = sex with non-steady partners.

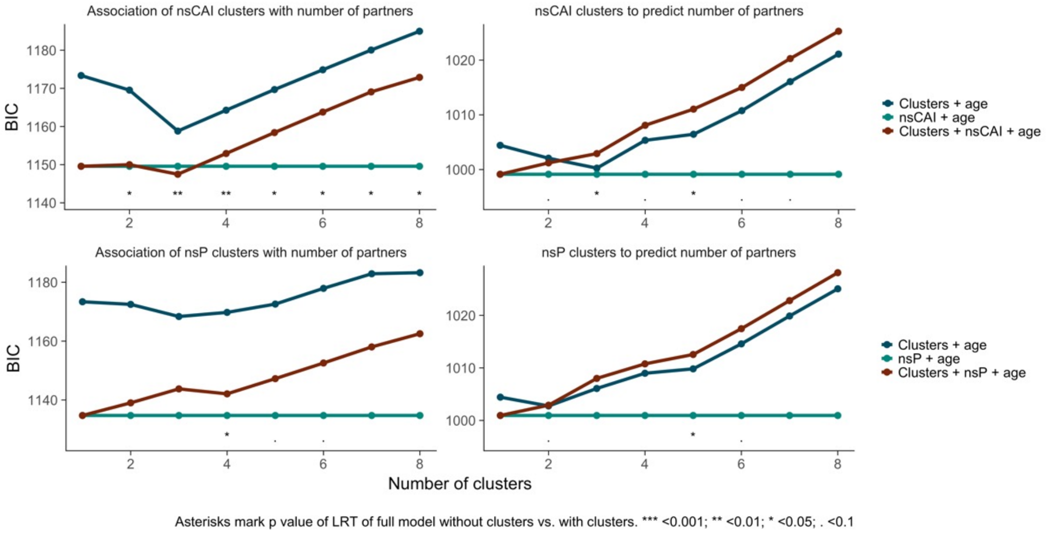

Fig N: BIC values for models to explain and predict number of sexual partners. Association of different numbers of clusters with number of sexual partners (left), and different numbers of clusters to predict number of sexual partners (right). For clusters based on nsCAI trajectories (above) or nsP trajectories (below). nsCAI/nsP as a predictor variable in models refers to nsCAI reported at time of reporting number of partners (left), or nsCAI reported before cut-off (right), respectively. A lower BIC represents a better prediction. Asterisks mark the p-value of the likelihood ratio test between the second and third model. BIC = Bayesian information criterion. LRT = likelihood ratio test. nsCAI = condomless anal intercourse with non-steady partners. nsP = sex with non-steady partners.
