# Supplementary material for: Unsupervised machine learning predicts future sexual behaviour and sexually transmitted infections among HIV-positive men who have sex with men
Source: PLoS Comput Biol. 2022 Oct 27;18(10):e1010559. doi: 10.1371/journal.pcbi.1010559 (PMC9642906; doi:10.1371/journal.pcbi.1010559)
Supplement: Attachment — Submitted filename: 20220620_ML_STIs_Reviewer_Reply.pdf [file pcbi.1010559.s002.pdf]

Dear Dr. Wertheim and Dr. Leitner

We would like to thank the reviewers for the helpful comments and suggestions. Please find our replies below. The reviewers comments are marked in cursive, and new passages in the manuscript are written in green font.

We thank you again for the interest in our study and hope that you find the amendments we made satisfactory to render our manuscript suitable for publication in PLoS Computational Biology.

Sincerely,

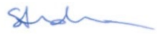

Sara Andresen

Reviewer #1: *The manuscript entitled "Unsupervised machine learning predicts future sexual behaviour and sexually transmitted infections among HIV-positive men who have sex with men" used statistical approaches to assess whether clusters (developed using a hierarchical clustering technique) enhance predictions of sexual behaviour or sexually transmitted diseases (STIs).*

Overall, I thought the paper read nicely and addressed an important policy question. However, I thought there were some methodological gaps. Below are the gaps that I think should be addressed. Thank you for letting me read your manuscript!

> Response: We would like to thank the reviewer for the positive assessment of our work.

Major comments

1. The authors used likelihood ratio test (LRT), AIC, BIC, and auROC to assess the predictive performance of the clusters. To my knowledge, LRT, AIC, and BIC are typically used for model selection and not prediction. While these three metrics showed that a model including the cluster variables improves model fit, these metrics do not assess prediction. Therefore, based on my understanding, statements such as those on lines 174 ("…improved the model fit for predicting") and 180 ("…improved model performance") are not accurate as LRT does not assess prediction.

> Response: We used regression to assess whether adding clusters to existing models would improve model fit. LRT was chosen as it can aptly compare the goodness of fit of nested models; and AIC and BIC were chosen for their over-/underfitting assessment properties. In other words, we compare a regression model for STIs that applies conventional variables to the same model, but augmented by the behavioural clusters. The aim of this study was to apply this clustering method (which can be seen as a dimensionality reduction framework for time-varying data) to a clinical routine problem rather than to find the ideal way to predict STIs within the Swiss HIV Cohort Study. We however acknowledge that more accurate wording could help the interpretation of our results. Therefore, in line with the reviewer's comment, we further clarify this in the revised manuscript. The relevant section in the methods now reads:
>
>> We used likelihood ratio tests (LRT) and Bayesian information criteria (BIC) to compare model fits with and without behavioural clusters. Likelihood ratio tests were chosen as they can aptly compare the goodness of fit of nested models, while Bayesian information criteria were chosen for their over-/underfitting assessment properties.
>
> And we added the following passage in the discussion:

We recognise that there may be more performant ways to predict STIs. However, the aim of this study was to test the relevance of behavioral clusters for understanding the epidemiology of STIs (i.e. to assess whether these clusters were associated with distinct patterns of STI incidence) rather than to find the ideal way to predict STIs within the SHCS. We test the association between behavioral clusters and STI in a predictive context but, more generally, this analysis also informs about which dimensions of human behavior matter most for STI incidence and how complex temporal variation of behavioral data can be best simplified to capture these essential dimensions.

2. The manuscript did not discuss the use of a training and validation datasets. As written, it seems that the entire dataset was used to assess prediction. Without the use of training/validation datasets the prediction is typically too optimistic. Creating training/validation datasets seem to be the standard approach for prediction, so it would be nice to understand why that was not used.

Response: We did not originally consider training and validation datasets because our analysis was not a predictive one in the classic sense (please see our response to comment 1). Further, we did not expect there to be a large risk of overfitting given the small number of parameters and the large number of events. However, following the reviewer's suggestion, we now added an analysis using a 5-fold cross-validation in the supplementary of the revised version. Accuracy in the test set ranged between 78% and 91% for predicting future sexual behaviour and STIs. As seen in the ROC analysis, adding clusters to a model considering other predictors brought only marginal and in some cases no benefit in accuracy. We report these analyses in the revised supplementary material (see Figures S4 and S5).

3. The paper makes that claim that the use of the clusters increases the prediction. However, it would be nice to see a more robust model selection framework, i.e., including the previous two nsCAI values as variables (instead of just the previous one) or an "ever nsCAI" variable as well as investigation of the functional form of age.

Response: Following the reviewers' suggestion, the revised version contains analyses including the previous two values, an "ever reported nsCAI" variable, a "mean nsCAI" variable. The results suggest that considering clusters consistently yields a better model performance than using an "ever reported nsCAI" variable, though in most cases a worse model performance than using the last two nsCAI values available, or a "mean nsCAI" value. Considering the last two nsCAI values available strongly improved model performance for predicting future nsCAI, yet yielded little to no performance improvement for predicting future STIs and syphilis. We used age as a linear predictor as exploring its functional form showed a steady decrease of STI incidence with age. We display the analyses above in the revised supplementary material (see Figures S2 and S6) and added the following passage to the results section:

Comparing models with behavioural clusters to models including other metrics derived from past behaviour showed that while clusters improve model fit, equal or better improvements can be achieved by considering other parameters, such as the last two available nsCAI values, or using a mean nsCAI value before cut-off. Models considering behavioural clusters performed consistently better than those only considering whether a participant had ever reported nsCAI (Supplementary Figure S6).

4. The auROC metric (which was the one metric in the paper that I typically have seen used to assess prediction) did not seem very different with and without the clusters (as seen in Table 2). This left me wondering if the unsupervised machine learning clusters really did increase prediction. Especially once training and validation datasets are created and a more robust model selection approach is taken.

Response: The areas under the receiver operator characteristic curve were consistently higher for models including clusters, though the differences were small (See Table 2). Please also see our responses to points 1 and 2.

Minor comments

1. Not sure if "corrected our models" is the right term; maybe use control or adjust (see line 108 for an example).

Response: Thank you for pointing this out. We now use "adjust" consistently.

2. I was not clear on lines 281-284 regarding the sexual contact network. It would be helpful to include more details on the connection between the analysis and contact networks.

Response: We elaborated on this point. The section of the discussion now reads:

> Another explanation could be that clustering individuals based on longitudinal behaviour data identifies sexual contact networks, i.e. that individuals with similar behaviour over time may be more likely to have sex together, thus explaining similar STI incidence (22). A recent proof of concept study explores this possibility using HCV phylogenies (23).

Reviewer #2: *Thank you for giving me the opportunity to review this manuscript. I thought it was well written and the subject is interesting. Although the subject of machine learning is not my expertise, I do have some minor points that will hopefully help to further improve manuscript.*

Response: We would like to thank the reviewer for the positive assessment of our work.

Abstract
- 2nd paragraph: I find "up to a certain cut-off point" rather vague. I would suggest just to provide the cut-off date here.

Response: We thank the reviewer for this comment. We now mention the cut-off date explicitly.

- I am not sure what the author mean with the last paragraph of the abstract and I do not think this is elaborated on the Discussion section of the main paper. Do you mean the clusters with "framework"? And how can this framework be used as an alternate method for categorization (this is also mentioned in the Conclusion of the main paper) and how can it contribute to a better understanding of time-varying risk factors?

Response: By "framework", we mean the methodology of generating and evaluating the clusters. For clarity, we changed it accordingly in the abstract. Time-varying variables often enter statistical models as "ever [had sex without a condom]" or "recently [had sex without a condom]". We believe there is a wealth of information in the trajectory of a time series, though this data is often very high-dimensional. We propose hierarchical clustering as a means to distil information from these time series into a single variable (in our case, cluster membership). This variable could define groups of people likely to change behaviour simultaneously, in turn aiding projections.

Methods:
- Line 59: how did you define a non-steady partner? Or a steady partner?

Response: This is the original wording from the follow-up questionnaire, in which the terms are unfortunately not further specified. Therefore, there are certainly different interpretations of these terms. We recognise that this is a source of uncertainty and potential bias.

- Line 65: suggestion to refrain from using abbreviations that are not commonly used such as "nsCAI" and "nsP" if the word count allows it. This would increase the readability of the manuscript.

Response: In line with the reviewer's suggestion, we replaced the abbreviations by the full definition in the discussion section. However, we decided to keep the abbreviations for the methods and results sections, since they make reporting easier (for the figures in particular).

Results:
- Figure 1: panels for step 1 and 2 are rather small now and the text was very hard to read. Would this be possible to increase the size?

Response: Thank you for your comment. We increased the font size in Figure 1.

- Why was only syphilis routinely tested and not chlamydia and gonorrhea?

Response: Testing for STIs was performed according to the standards defined by the Clinical and Laboratory Committee (CLC) of the Swiss HIV Cohort Study (www.shcs.ch). During the study period, the CLC decided to include serial screening for syphilis, but not for chlamydia and gonorrhea. This decision was based on considerations regarding epidemiological trends, feasibility and practicability in the SHCS.

- Table 1: could you explain what is considered "mandatory schooling" in Switzerland? Is that similar to primary school and high school for example? And what school level corresponds to "finished apprenticeship?"

Response: Mandatory schooling in Switzerland runs from grade one through nine. A finished apprenticeship would typically follow after these nine years of schooling and include three or more years of practical on-the-job training in combination with part-time classroom schooling. We now state this in the caption of table 1.

- Figure 2: I am not sure if the "total" line is necessary in this figure

Response: We decided to keep the total line so that it is possible to compare a single cluster's trajectory to the average.

Discussion:
- Line 284: do you mean the sensitivity analysis last mentioned in the Results section here? If so, I would clarify this.

Response: Thank you for pointing this out. We now specify this explicitly.

- Line 292: Indeed, asymptomatic STIs may likely go unnoticed and thereby impact your outcome if it is based on self-report. How do you think this would impact your results if, instead of self-report, the outcome was based on actual STI testing done at follow-up visits?

Response: Thank you for this excellent question. One could hypothesise that since both the variable determining cluster membership (sexual behaviour over time) and the outcome (STIs) are self-reported, there may be a stronger correlation mediated by reporting bias (i.e. participants reporting more condomless sex being systematically more likely to also self-report STIs). Additionally, participants with similar sexual behaviour over time may also have similar STI testing habits, thus also potentially leading to a stronger correlation between clusters and self-reported outcomes. This hypothesis is supported by the finding that the clusters enhance predictions for self-reported STIs more than for syphilis, which is based on serological testing. We added a line on this in the discussion:

> It is conceivable that the stronger association between cluster membership and nurse/physician-reported STIs compared to laboratory-confirmed syphilis results from similar testing and STI reporting behaviour amongst individuals in the same behavioural cluster.
